# Simple Structural Differences between Coding and Noncoding DNA

**DOI:** 10.1371/journal.pone.0014651

**Published:** 2011-02-03

**Authors:** Kenneth J. Locey, Ethan P. White

**Affiliations:** 1 Department of Biology, Utah State University, Logan, Utah, United States of America; 2 The Ecology Center, Utah State University, Logan, Utah, United States of America; London School of Hygiene and Tropical Medicine, United Kingdom

## Abstract

**Background:**

The study of large-scale genome structure has revealed patterns suggesting the influence of evolutionary constraints on genome evolution. However, the results of these studies can be difficult to interpret due to the conceptual complexity of the analyses. This makes it difficult to understand how observed statistical patterns relate to the physical distribution of genomic elements. We use a simpler and more intuitive approach to evaluate patterns of genome structure.

**Methodology/Principal Findings:**

We used randomization tests based on Morisita's Index of aggregation to examine average differences in the distribution of purines and pyrimidines among coding and noncoding regions of 261 chromosomes from 223 microbial genomes representing 21 phylum level groups. Purines and pyrimidines were aggregated in the noncoding DNA of 86% of genomes, but were only aggregated in the coding regions of 52% of genomes. Coding and noncoding DNA differed in aggregation in 94% of genomes. Noncoding regions were more aggregated than coding regions in 91% of these genomes. Genome length appears to limit aggregation, but chromosome length does not. Chromosomes from the same species are similarly aggregated despite substantial differences in length. Aggregation differed among taxonomic groups, revealing support for a previously reported pattern relating genome structure to environmental conditions.

**Conclusions/Significance:**

Our approach revealed several patterns of genome structure among different types of DNA, different chromosomes of the same genome, and among different taxonomic groups. Similarity in aggregation among chromosomes of varying length from the same genome suggests that individual chromosome structure has not evolved independently of the general constraints on genome structure as a whole. These patterns were detected using simple and readily interpretable methods commonly used in other areas of biology.

## Introduction

Evidence that selection affects the organization of information within genomes has resulted in efforts to characterize large-scale patterns of genome structure. Recently, advanced statistical and graphical methods such as chaos game theory, wavelet analyses, information theory, thermodynamics, and fractal geometry have been used to examine large-scale genome structure [1]–[12]. The results of these studies have increased our knowledge of how genomes are organized by moving beyond simple characterizations such as genome length and GC content, to study how the distribution and organization of information within genomes may be evolutionarily constrained [7]. While statistically informative, the structures quantified by these studies can be difficult to understand, making it difficult to interpret how the observed statistical patterns relate to the physical distribution of genomic elements.

Considering the difficulty of linking complex statistical patterns to the physical structure and biological processes affecting genomic evolution, we ask whether patterns in large-scale genomic structure can be quantified using a simpler approach with an intuitive structural interpretation. This simplification has the potential to allow for less statistically abstracted interpretations of genomic structural patterns. Here, we attempt such an approach using a straightforward definition of one of the most intuitive structural properties of sequential data, aggregation. We use this measure to detect a general difference among the two major kinds of DNA and the two forms of nitrogenous bases commonly used in other studies [1], [4], [6], [8], [13]–[14]. Specifically, genomes are comprised of regions of DNA that code or do not code for proteins and are composed of two different structural forms of nitrogenous bases, purines (Pu) represented by adenine and guanine, and pyrimidines (Py) represented by thymine and cytosine. Assuming that coding and noncoding DNA are structured by different selective forces [14], common units of coding and noncoding regions (i.e. Pu and Py) may exhibit different distributions resulting from different structuring forces. Our aim was to use Morisita's Index of aggregation (I_M_) [15]–[18] to examine whether: 1) Pu and Py exhibit non-random structure within sequences; 2) aggregation differs between coding and noncoding DNA; and 3) patterns of aggregation differ among chromosomes of the same species and among taxonomic groupings. If meaningful patterns can be detected this suggests that aggregation may provide an intuitive measure of structural genomic patterns that can be meaningfully influenced by biological processes.

## Results

**Figure 1:**
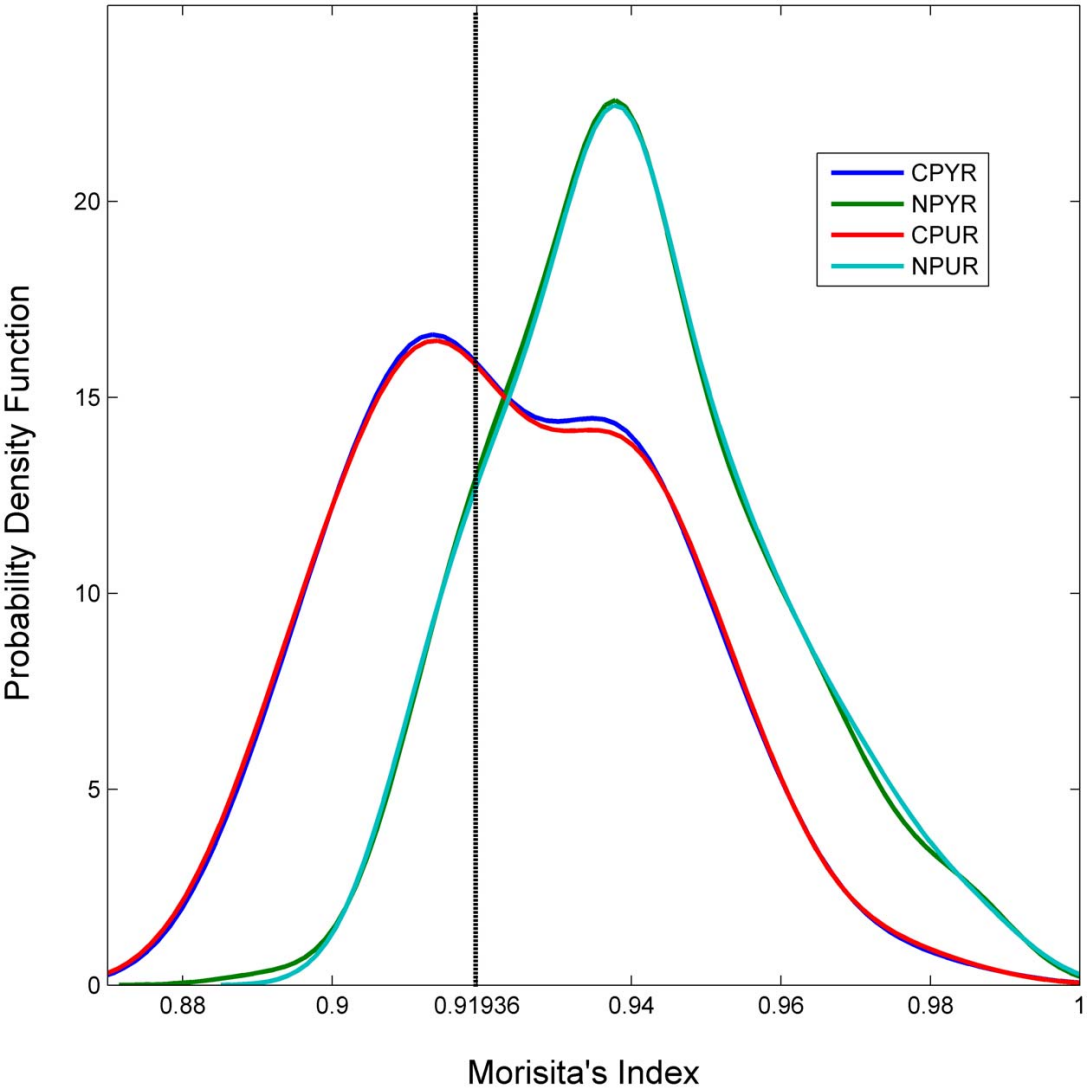
Kernel density curves reveal different distributions for coding and noncoding DNA. Kernel density curves for purines and pyrimidines within coding (C) and noncoding DNA (N). Distributions for purines and pyrimidines nearly completely overlap. Curves for noncoding DNA are shifted towards higher values of aggregation while curves for coding DNA are centered closer to the derived value for randomness, 0.91936. Apparent bimodality within coding regions may have resulted from the sample-size of different taxonomic groupings (e.g. 32 Gammaproteobacteria within a narrow range), but note the lack of bimodality among corresponding noncoding regions of the same set of genomes.

Purines (Pu) and pyrimidines (Py) were distributed similarly within genomes and chromosomes, as illustrated by nearly identical distributions within coding and noncoding DNA (Fig. 1) and the similar results of statistical analyses (Table 1 and 2). In coding DNA Pu and Py were less aggregated (i.e. more evenly distributed) than random in approximately 44% of genomes, and more aggregated than random in almost 52% of genomes (p<0.01; Table 1). Noncoding DNA was rarely more evenly distributed than random (∼10% of genomes) with 86% of genomes exhibiting significant aggregation (p<0.01; Table 1). The difference in aggregation between coding and noncoding DNA was significant in 94% of chromosomes (n = 245). Of these 245 chromosomes, noncoding DNA was more aggregated than coding DNA in 91% of cases (n = 224). Hence, coding DNA was more aggregated than noncoding DNA in only 21 chromosomes (8.0%), from 18 genomes.

**Table 1:** Aggregation among microbial genomes.

Genomes, N = 223	Coding		Noncoding	
	Pu	Py	Pu	Py
Aggregated	52.0% (n = 116)	52.0% (n = 116)	86.1% (n = 192)	86.1% (n = 192)
Random	5.4% (n = 12)	4.0% (n = 9)	3.6% (n = 8)	4.0% (n = 9)
Overdispersed	42.6% (n = 95)	44.0% (n = 98)	10.3% (n = 23)	9.9% (n = 22)

**Table 2:** Aggregation among microbial chromosomes.

Chromo, N = 261	Coding		Noncoding	
	Pu	Py	Pu	Py
Aggregated	46.7% (n = 122)	47.9% (n = 125)	80.4% (n = 210)	80.5% (n = 210)
Random	5.4% (n = 14)	5.4% (n = 14)	5.4% (n = 14)	5.7% (n = 15)
Overdispersed	47.9% (n = 125)	46.7% (n = 122)	14.2% (n = 37)	13.8% (n = 36)

Of the 18 genomes (21 chromosomes) where coding DNA was more aggregated than noncoding DNA, seven genomes belong to the Spirochaetes group. The other 11 genomes are widely distributed across groups: Alphaproteobacteria (3), Aquificae (1), Bacterioides/Chloribi (1), Betaproteobacteria (1), Crenarcheota (2), Euryarchaeota (1), Gammaproteobacteria (1), and Nanoarcheota (1). Only two of the 13 Spirochaete members represented in the dataset showed greater average aggregation in noncoding DNA than coding DNA. Compare this to Actinobacteria (N = 17), Thermotogae (N = 8), Firmicutes (N = 15), and Epsilonproteobacteria (N = 9) where all members showed greater average aggregation in noncoding DNA, or to Gammaproteobacteria (N = 32), Euryarchaeota (N = 11), or Betaproteobacteria (N = 26) where all but one member showed greater average aggregation in noncoding DNA. All other groups had three or fewer members lacking greater average aggregation within noncoding DNA than coding DNA. Hence, Spirochaetes appear to be the only phylum-level group where noncoding DNA is not typically more aggregated than coding DNA.

**Figure 2:**
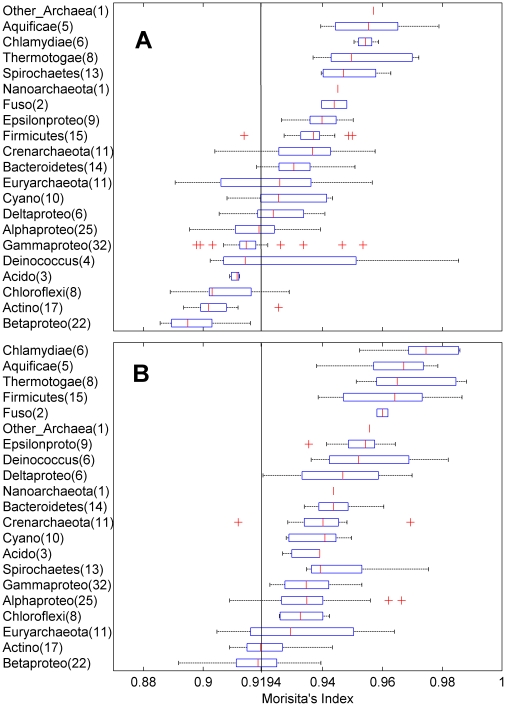
Box plots showing ranges of aggregation values (I_M_) for pyrimidines within coding and noncoding DNA of 21 microbial groups. The distribution of box plots for coding DNA (A) is shifted more towards lower values of aggregation and closer to randomness than those for noncoding DNA (B) which are shifted towards values of higher aggregation.

Aggregation varied significantly among phyla, with individual groups of taxa typically occupying narrow ranges of aggregation and having little-to-no overlap with most other groups (Fig 2). However, the distribution of taxonomic groups across the observed range of aggregation revealed no apparent phylogenetic clustering or pattern. For instance, proteobacteria are distributed throughout while archaeal groups are separated by bacterial groups. When the set of 200 genomes was examined as a group, with an average measure of aggregation for each genome represented by a single data point, coding and noncoding regions formed different distributions of aggregation with noncoding regions shifted towards higher values of aggregation (Fig. 1). Despite a smooth unimodal distribution of aggregation values among noncoding DNA, coding DNA from the identical set of genomes exhibited an apparent bimodality. While the first mode could be the result of sample bias, the lack of a corresponding mode in the curve for noncoding DNA suggests two different subgroups of genomes with aggregated noncoding DNA; one where the distribution of nitrogenous bases in coding DNA is under-aggregated to essentially random (I_M_ = 0.91936), and one where the distribution is significantly aggregated.

**Figure 3:**
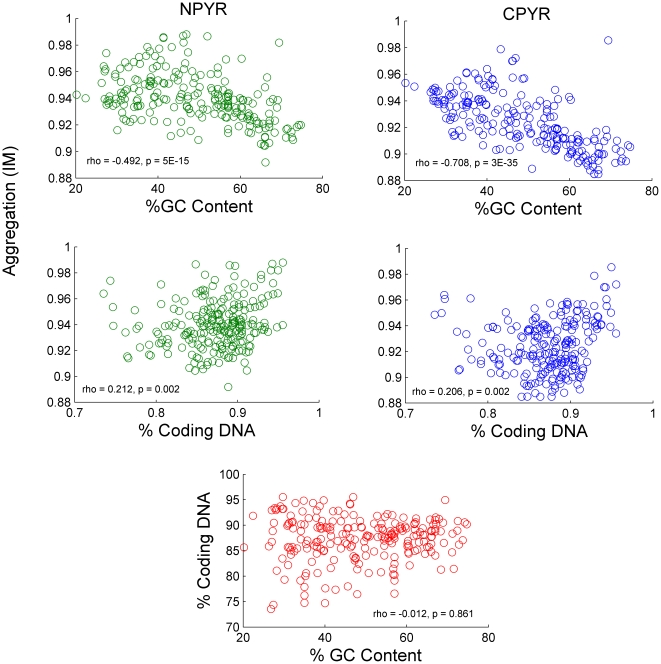
Plots of aggregation (I_M_) vs. % GC content and % coding DNA, with a plot of % coding DNA vs. % GC content. Aggregation of pyrimidines within coding DNA (blue) and noncoding DNA (green) shows a greater linear relationship to %GC content than to % Coding DNA. % Coding DNA and % GC (red) content are not correlated.

**Figure 4:**
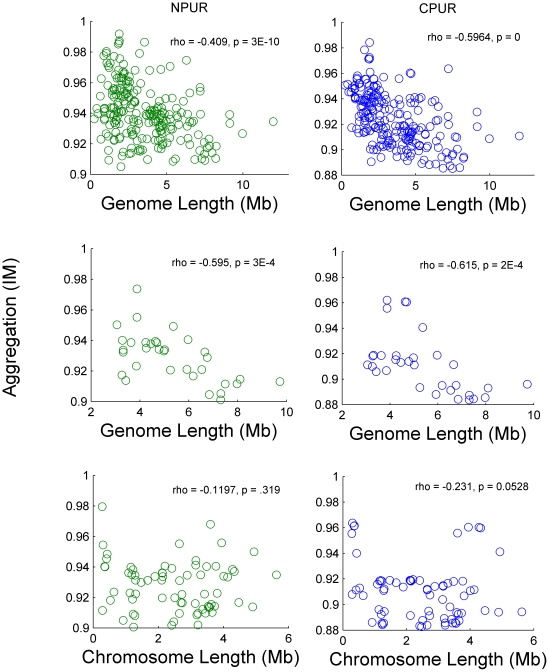
Plots of aggregation (I_M_) vs. genome length and chromosome length for Purines (Pu). (Top) Aggregation of purines in coding (blue plots) and noncoding (green plots) DNA for 223 genomes. (Middle) Aggregation of purines in coding and noncoding DNA for the 33 genomes with multiple chromosomes. (Bottom) Aggregation of purines in coding and noncoding DNA for 71 individual chromosomes from the 33 genomes with multiple chromosomes. These plots reveal that dissecting a genome into its constituent chromosomes destroys the generally decreasing pattern of aggregation with increasing genome length.

Aggregation, as estimated with Morisita's Index, showed a significant correlation with GC content and a slight but also significant correlation with percent coding DNA (Figure 3). Aggregation was also significantly correlated with genome length. The strength of the correlation and the shape of the distribution reveals that estimates of I_M_ decreased and converged on lower values with increasing genome length (Fig. 4), suggesting that larger genomes tend to be less aggregated. Among genomes with multiple chromosomes, I_M_ and chromosome length were not correlated (Fig. 4). However, when the lengths of these chromosomes were summed to obtain the length of the genome, the pattern of limited aggregation with increasing genome length was again obtained (Fig. 2). Additionally, aggregation was similar among chromosomes of the same species (average % difference = 0.28±0.04 SE for Py to 0.27±0.04 SE for Pu) despite large differences in chromosome length (average % difference = 91.3±9.23 SE).

## Discussion

Both structural forms of nitrogenous bases clearly exhibit non-random distributions within genomic sequences and are nearly always distributed similarly. Steps taken to remove statistical effects of density, sampling scale, and GC bias, and to examine the statistical relationships of aggregation to GC content, percent coding DNA, and genome length reveal that the reported differences between coding and noncoding DNA are likely due to meaningful patterns of Pu and Py clustering within sequences and not due to the statistical effects of these other variables. Despite removing statistical effects of GC-content by recoding genomes in Purines and Pyrimidines, and using a measure of aggregation that is independent of the ratio of coding to noncoding DNA, GC-content, genome length (but not chromosome length), and percent coding DNA were significantly correlated with aggregation. Though these results suggest that relationships among these structural genomic features are real, further studies will be necessary to understand these patterns.

Genome length appears to set a maximum limit on the degree of aggregation possible (Fig. 4). This pattern holds for genomes with single and multiple chromosomes. However, the lengths of chromosomes from multi-chromosomal genomes do not appear to show the same relationship. Instead, chromosomes of the same species are similarly aggregated despite large differences in length. When the lengths of these chromosomes are summed to obtain overall genome length, their summed lengths follow the decreasing pattern shown for single chromosome genomes (Fig. 4). At the chromosome scale, aggregation appears to be a property of the species, largely invariant with chromosome length. However, overall aggregation seems to be limited by genome length, perhaps regardless of the number of chromosomes comprising a genome. Both similarity in aggregation among chromosomes of varied length from the same genome, and the tendency for aggregation among chromosomes to be influenced by overall genome length, suggests that chromosome structure has not evolved independently of general constraints on overall genome structure.

Noncoding DNA was almost always more aggregated than coding DNA. In other words, nitrogenous bases of similar structure are more likely to be found in close proximity within noncoding DNA than within coding DNA. This conclusion is based on the genome-wide averaging of tens of thousands of estimates of I_M_ across a diverse collection of 223 microbial genomes, and hence, represents a general low-resolution pattern of genome structure. It may be unlikely that such a pattern is the result of one or even a few specific genetic or evolutionary processes. What it does suggest is that the functions that coding and noncoding DNA perform, and the pressures that affect their evolution, are different enough to manifest a general difference in the gross distribution of their common elements.

For Spirochaetes, the pattern is typically reversed. Spirochaetes are a small and cohesive group of gram-negative chemoheterotrophs. They are unusual in their linear chromosomes, cytoskeleton, long helical cells, and coevolution with a host-specific phage. As such, it is possible that these traits that distinguish Spirochaetes from other microbes explain their exception to the general pattern. However, a superficial investigation of the microbial traits is unlikely to explain this reversed pattern, because a variety of cell shapes (e.g. coccus, rod, spiral), chromosome shapes (e.g. linear, circular), temperature ranges (e.g. mesophilic, thermophilic), habitats (e.g. soils, sulfur springs, hosts), chromosome lengths (490885-5566749), and percent coding DNA (0.7475-0.9483), are represented within the set of 18 genomes where coding DNA was on average more aggregated that noncoding DNA.

The observed bimodality in the distribution of aggregation values for coding DNA suggests the presence of two general groups of genomes differing characteristically in the patterns of aggregation within coding DNA. Whether these two groups differ in a biologically meaningful way that influenced the distribution of structurally different nitrogenous bases has not yet been determined. Further investigation is necessary to determine whether this bimodality results from the specific genomes chosen for analysis or whether it is an indicator of an important biological process that has shaped genome evolution among microbes.

The distribution of phyla across the range of aggregation in this study strongly corroborates the pattern described by Bohlin et al. (2009) who examined the genomic fraction of purine and purine/pyrimidine stretches (i.e. an indirect measure of aggregation) in relation to environmental variables across a similar but smaller set of prokaryote phyla [19]. Though there are no methodological similarities, and noncoding DNA is analyzed separately from coding DNA in this study, both studies reveal that phyla occupy similarly ordered and narrow ranges of aggregation (Table 3). When comparing the ranks of phyla common to both studies there were four exact matches and four instances where phyla differed by only one rank. The reproduction of this pattern in spite of minimal methodological similarity suggests that the pattern is robust and relatable to functional traits that interface with the exogenous environment (Bohlin et al. 2009).

**Table 3:** Phyla ranked according to aggregation of purines, averaged for coding and noncoding DNA, as reported here, and as reported in the results of Bohlin et al. (2009).

	Present Study	Bohlin et al. (2009)
Rank	Purine Aggregation	Purine Stretches
1	Chlamydia	Thermotoga
2*	Thermotoga	Spirochaetes
3	Firmicutes	Chlamydia
4	Spirochaetes	Euryarcheota
5	Deltaproteo	Crenarchaeota
6*	Crenarchaeota	Firmicutes
(7)	Epsilonbacteria	Epsilonbacteria
8*	Cyanobacteria	Deltaproteo
9	Alphaproteo	Cyanobacteria
(10)	Gammaproteo	Gammaproteo
11	Euryarcheota	Chloroflexi
12*	Chloroflexi	Alphaproteo
(13)	Actinobacteria	Actinobacteria
(14)	Betaproteo	Betaproteo

Ranks in parentheses (n = 4) are exact matches, ranks with asterisks (n = 4) are one rank different.

Despite the potential for exceedingly complex distributions of bases within coding and noncoding regions, the study of large-scale genomic structure clearly does not preclude the use of simple approaches to arrive at general patterns based on intuitive properties. It is clear that those forces that have structured protein coding and noncoding regions, as well as individual chromosome and overall genome structure, have left evidence of their effects at the level of common elements, the two types of structural nitrogenous bases. We suggest that processes and constraints with predominant effects on genome structure should influence the patterns of aggregation observed in this study. While statistical approaches to large scale genome structure have the potential to reveal novel and meaningful patterns as well as structural relationships, we suspect that the general patterns reported here are unlikely to be explained by statistical approaches alone, that is, without establishing the genetic or evolutionary mechanisms. Lack of clarity in the interpretation of statistical methods, metrics, and results that document novel and poorly understood structural patterns can only be a detriment to this endeavor.

## Materials and Methods

### Obtaining genomic data

We created Perl scripts to examine 261 chromosomes of 223 genomes from 21 phylum level microbial groups, downloaded from the National Center for Biotechnology Information microbial genome website, www.ncbi.nlm.nih.gov/genomes/lproks.cgi. We downloaded FASTA sequence and GenBank feature files. We picked genomes and chromosomes that represented a broad range of lengths and protein coding contents. Pearl scripts (Program Script S1 & S2) and a table of microbial genome information and per chromosome results (Table S1) can be accessed through supplementary materials.

### Genome handling and aggregation estimation

We obtained estimates of aggregation for coding and noncoding DNA by using a sliding window approach to estimate the average aggregation of Pu and Py among consecutive non-overlapping 100-base sections of chromosomes. Rather than examine each individual coding or noncoding region separately, we examined coding and noncoding DNA as concatenated sequences of individual regions. These approaches alleviated two problems. First, analyzing individual coding and noncoding regions leaves a considerable amount of genome unanalyzed because individual coding and noncoding regions are rarely perfect multiples of a particular window size. Second, information regarding GC content is lost when sequences are binarily recoded according to Pu (A,G) and Py (C,T), hence removing potential statistical effects of GC content on aggregation.

We used Morisita's Index (I_M_) [15]–[18] as our aggregation metric. I_M_ is commonly used in ecological and evolutionary studies [20]–[23] to study the spatial distribution of age classes, genotypes, and species, and has been shown to be a more precise and less biased descriptor of spatial aggregation than other methods (e.g. variance∶mean ratio) [15]. I_M_ uses the number of occurrences among subsections of sampling areas (i.e., windows) to estimate measurements of aggregation based on a sampling probability. Specifically, I_M_ measures how many times more likely it is that two randomly selected individuals will be from the same subsection of study area than if the individuals in the population were distributed at random. For example, I_M_ = 1.5 indicates that the probability of sampling two individuals from the same quadrat is 50% greater than if the population was randomly distributed (i.e., Poisson distributed). An I_M_ of 0.5 indicates this probability is 50% less likely than random. I_M_ is not typically used in cases of severely limited occupancy (e.g. linear segments of genomes of *n* size holding, at most, *n* Pu or Py). As a result, the value representing randomness was offset from I_M_ = 1.0 to I_M_ = 0.91936 (SE = .000057), as determined from 20,000 randomizations. Therefore we compared observed values to randomizations of the same sequence (see below) to determine if the genome was more of less aggregated than random and to determine whether or not this difference was statistical meaningful.

Morisita's Index is calculated as:
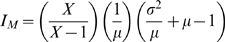
where X is the total number of individuals in the sampling universe, *μ* is the mean number of individuals per quadrat (i.e. subsection of the sampling universe), and *σ* ^2^ is the variance of individuals among quadrats. The formulation here is identical to that in Hurlbert (1990). In the present study, X is the total number of Pu (or Py) in a 100 base section of a genome, referred to here as a window, μ is the average number Pu or Py within each 10 base subsection of the window, and *σ* ^2^ is the variance of Pu or Py among the 10 subsections. It can be seen from the above equation that Morisita's Index is independent of genome length, genome segment length, and number of genome segments and is thus independent of the density of individuals in the window [15]. Using I_M_ thus controlled for differences in the density of Pu and Py among genomes. We also confirmed that I_M_ was insensitive to window and subsection size by reanalyzing a random subset of 29 genomes using several combinations of window size (100, 400) and subsection size (10, 20, 40). These combinations yielded qualitatively similar results (see table in supplementary materials).

### Randomizations

We created 100 randomized versions of each genome for comparison with actual genomes by randomly redistributing Pu and Py within individual coding and noncoding regions. These randomized genomes were analyzed as described above for comparison to actual genomes. By avoiding changes in the number of Pu and Py among individual regions, observed differences reflect the effect of nitrogenous base order; another control for the effects of Pu and Py density. P-values were determined to be less than 0.01 when average measurements of I_M_ from real genomes were greater than those from all 100 randomizations or less than those from all 100 randomizations.

### Statistical analysis

Microbial genomes typically contain a much larger fraction of coding than noncoding DNA. Here, the percentage of coding DNA ranged from 73.54 to 95.54%. Under this circumstance, I_M_ is calculated more times for coding DNA (typically tens of thousands) than noncoding DNA (typically thousands). To account for this difference in sample size, we chose non-parametric rank-sum tests to determine whether Pu and Py generally differ in aggregation between coding and noncoding regions of individual genomes. Additionally, we conducted Spearman's rank correlation to determine whether aggregation was related to percent coding DNA, genome length, chromosome length, and GC-content. We chose a nonparametric correlation technique because all datasets were non-normally distributed as determined from the Lilliefors test for normality. We used the student version of MATLAB v7.7.0 to generate kernel density curves, box plots, and to conduct all statistical analyses.

## Supporting Information

Table S1 This table list those microbes used for analysis in this study. Results for rank-sum tests and average calculation of Morisita's Index of aggregation are presented in following columns (N = noncoding, C = coding, Pur = purine, Pyr = pyrimidine).
(0.18 MB XLS)

Program Script S1 A plain text document of the script named genomic_agg, created by Ken Locey. This script is to be run after the gff_reader script.
(0.02 MB TXT)

Program Script S2 A script to be run before genomic_agg. This script uses Genbank and Fasta files, checks them for agreement, and generates a file used by genomic_agg. This script was created by Ken Locey.
(0.00 MB TXT)
